# Supplementary material for: MAPK15 Prevents IFNB1 Expression by Suppressing Oxidative Stress-Dependent Activation of the JNK-JUN Pathway
Source: Int J Mol Sci. 2025 May 27;26(11):5148. doi: 10.3390/ijms26115148 (PMC12155439; doi:10.3390/ijms26115148)
Supplement: Supplementary file 1 [file ijms-26-05148-s001.zip › Figure S1.pdf]

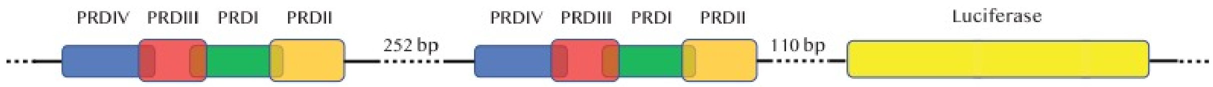

Figure S1. Schematic representation of the human *IFNB1* promoter in IFN-Beta_pGL3 plasmid. The figure shows the four classical regulatory domains, PRDI-PRDIV, which serve as binding sites for transcription factors involved in *IFNB1* gene regulation. Specifically, PRDIV is bound by AP-1 factors; PRDIII and PRDI are recognised by IRF3/IRF7 proteins; PRDII is the binding site for NF-κB. The figure also indicates the distances, in base pairs (bp), between these elements and their position relative to the luciferase reporter gene.
