# Supplementary material for: MAPK15 Prevents IFNB1 Expression by Suppressing Oxidative Stress-Dependent Activation of the JNK-JUN Pathway
Source: Int J Mol Sci. 2025 May 27;26(11):5148. doi: 10.3390/ijms26115148 (PMC12155439; doi:10.3390/ijms26115148)
Supplement: Supplementary file 1 [file ijms-26-05148-s001.zip › Figure S2.pdf]

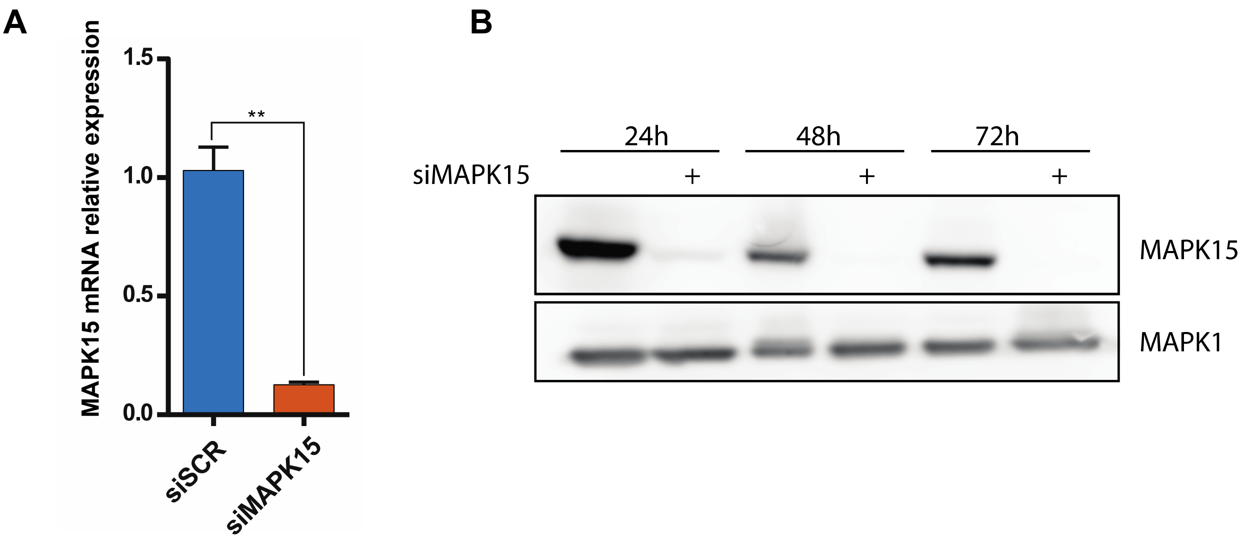

Figure S1. Validation of *MAPK15* silencing. (**A**) RT-qPCR analysis of *MAPK15* mRNA expression in 16HBE14o- cells transfected with control scramble siRNA (siSCR) or siRNA against MAPK15 (siMAPK15), for 72h. (**B**) Western blot analysis showing levels of MAPK15 protein in 16HBE14o- cells, transfected with control scramble siRNA or siRNA against MAPK15, for 24, 48 and 72h.
