# Supplementary material for: MAPK15 Prevents IFNB1 Expression by Suppressing Oxidative Stress-Dependent Activation of the JNK-JUN Pathway
Source: Int J Mol Sci. 2025 May 27;26(11):5148. doi: 10.3390/ijms26115148 (PMC12155439; doi:10.3390/ijms26115148)
Supplement: Supplementary file 1 [file ijms-26-05148-s001.zip › Table S1.pdf]

Gene	Primer	Sequence (5'→3')
MAPK15	Forward	TGGCCAGCGTACAACAGGT
	Reverse	CAGTCCCGTAGGCTTGGGAGTA
IFNB1	Forward	ACGCCGCATTGACCATCTATGAGA
	Reverse	ACTGCTCATGAGTTTTCCCCTGGT
IFIT2	Forward	GGAGCAGATTCTGAGGCTTTGC
	Reverse	GGATGAGGCTTCCAGACTCCAA
IFIT3	Forward	CCTGGAATGCTTACGGCAAGCT
	Reverse	GAGCATCTGAGAGTCTGCCCAA
MX1	Forward	GGCTGTTTACCAGACTCCGACA
	Reverse	CACAAAGCCTGGCAGCTCTCTA
CCL2	Forward	GATCTCAGTGCAGAGGCTCG
	Reverse	TGCTTGTCCAGGTGGTCCAT
CCL5	Forward	AGAGGATCAAGACAGCACGTGGAC
	Reverse	GGAGCACTTGCCACTGGTGTAGAA
CXCL8	Forward	GTTTTTGAAGAGGGCTGAG
	Reverse	TTTGCTTGAAGTTTCACTGG
IL6	Forward	GCAGAAAAGGCAAAGAATC
	Reverse	CTACATTTGCCGAAGAGC
B2M	Forward	TGGGTTTCATCCATCCGACA
	Reverse	GCGGCATCTTCAAACCTCCA

Table S1: Primer sequences
